# Anesthetic Management for Cesarean Delivery in a Patient With a Difficult Airway and Risks for Postpartum Hemorrhage: A Case Report

**DOI:** 10.7759/cureus.47428

**Published:** 2023-10-21

**Authors:** Bansi V Patel, Reine Zbeidy, Alexander Hall, Selina D Patel

**Affiliations:** 1 Anesthesiology, Hospital Corporation of America (HCA) Florida Osceola Hospital, Kissimmee, USA; 2 Department of Anesthesiology, Pain and Perioperative Medicine, University of Miami, Miami, USA; 3 Department of Anesthesiology, Jackson Memorial Hospital, Miami, USA

**Keywords:** case report, awake fibreoptic intubation (afoi), caesarean delivery (cd), postpartum haemorrhage (pph), difficult intubation, difficult airway

## Abstract

Neuraxial anesthesia is the preferred anesthesia technique for cesarean delivery, however, conversion to general anesthesia may be required for a variety of clinical scenarios, including massive post-partum hemorrhage. Obstetric patients are known to have otherwise more difficult airways and emergent situations can increase the likelihood of failed intubation with potentially disastrous consequences. We describe a novel anesthesia technique for cesarean delivery using neuraxial anesthesia as the primary anesthetic technique and electively securing the airway for a patient with multiple risk factors for post-partum hemorrhage and features concerning difficult intubation.

## Introduction

For cesarean deliveries (CD), post-partum hemorrhage (PPH) is defined as blood loss greater than 1000 ml within the first 24 hours of delivery and is a leading cause of maternal morbidity and mortality worldwide [1]. Risk factors for developing PPH include uterine atony, prior PPH, prior myomectomy, large fibroids, placenta previa, and placental abruption to name a few. During CD, utilizing neuraxial anesthesia techniques has been associated with less blood loss when compared to general anesthesia (GA). However, in the case of large to massive PPH (>1000 ml blood loss), conversion to general anesthesia may be necessary and being able to establish a secure airway quickly is of critical importance. Obstetric patients are known to have otherwise more difficult airways due to the anatomical and physiological changes of pregnancy, and when compounded with additional features of a difficult airway, it can pose a significant challenge for the anesthesiologist. This case report was written with the patient’s consent and was presented as a poster at the American Society of Anesthesiology Conference on October 9, 2021. It describes the novel anesthetic management of a patient presenting for an elective CD with multiple risk factors for PPH and features of an anticipated difficult airway.

## Case presentation

A 36-year-old G2P1001 presented for elective CD at 37 weeks of gestation due to a history of previous open myomectomy, previous CD and the presence of posterior placenta previa. The patient had multiple ultrasounds during pregnancy, with the most recent conducted at 36 weeks of gestation, none of which showed any concern for the morbidly adherent placenta. Her obstetric history was further complicated by the presence of multiple sub-serosal fibroids (largest 17 x 11 x 11 cm) and fetal macrosomia (fetal abdominal circumference (AC)>97%). Other pertinent features in her medical history included gestational diabetes A1 (A1GDM) and previous jaw surgery following trauma three years ago, requiring tracheostomy tube placement for two months. Her airway examination revealed a Mallampati 4 score with minimal mouth opening (<2 fingers) as her jaw had been surgically fixed bilaterally. She had a full range of motion of her neck but stated she sustained several new episodes of epistaxis in this pregnancy suggestive of airway edema. Urgent ENT consultation was sought, and flexible nasal endoscopy revealed a slightly swollen airway mucosa without any evidence of tracheal stenosis.

Premedication with sodium citrate 30 mL, metoclopramide 10 mg, dexamethasone 10 mg, and glycopyrrolate 0.2 mg were given. Vascular access included one 14G, two 16G peripheral intravenous lines, and a left radial arterial line. A lumbar epidural was placed at L4/L5 using a dural puncture epidural (DPE) technique. The airway was then secured using an awake fiberoptic technique via the right nostril. The airway was anesthetized with nebulized and topical lidocaine, and sedation was provided with a titrated remifentanil infusion. A 6.0 cm reinforced endotracheal tube (ETT) was inserted without any difficulty. After successful intubation, 15 ml 2% lidocaine with bicarbonate and 1:200000 adrenaline, and 50 mcg fentanyl were carefully titrated to achieve a T4 surgical anesthesia level using pinprick. A remifentanil infusion was titrated throughout surgery for endotracheal tube tolerance only; no general anesthesia was administered. The surgery commenced and a healthy baby was born 12 minutes after incision (Apgar score 9, 9, 9). The surgery was complicated with a PPH of 1500 ml requiring two units of blood transfusion, however, the patient remained hemodynamically stable throughout. The patient had a preoperative hemoglobin of 9.8 g/dl and her post-transfusion hemoglobin in the recovery area was 8.1 g/dl. An arterial blood gas revealed normal metabolic and respiratory parameters and a hematocrit of 23 L/L. Epidural morphine (3 mg) was administered after the baby was delivered for postoperative analgesia. The patient was kept intentionally awake and aware throughout the surgery and had full recall of the delivery. She was extubated at the end of the case and was extremely satisfied with her anesthetic care.

## Discussion

This is the first published case report (to our knowledge) that describes performing CD using neuraxial anesthesia as the primary anesthetic technique with an electively secured airway. This novel technique allowed the parturient to avoid GA (and the inherent risks associated), minimize blood loss, be awake during the birth of her baby as well and provide optimal postoperative analgesia with neuraxial morphine.

Our patient had multiple risk factors for PPH including previous uterine surgery, fibroids, placenta previa, and fetal macrosomia. As such, we were prepared with multiple large-bore intravenous access and blood products to be available in the operating room prior to incision. Similarly, invasive arterial line placement was also established for close hemodynamic monitoring and serial blood sampling in the event of significant PPH.

Both general anesthesia and neuraxial anesthesia techniques have been successfully used for CD complicated with maternal hemorrhage. The decision on the choice of anesthetic technique should be individualized, evaluating the risks and benefits specifically for each patient. We chose to use neuraxial anesthesia as our primary anesthetic technique as it avoided GA exposure to the baby (which could have been considerable given the patient’s previous surgical history), allowed the mother to be awake for the delivery, and provided optimal postoperative analgesia with epidural morphine. Furthermore, GA has been identified as a risk factor for postpartum hemorrhage during CD [2] and is associated with higher blood loss when compared with neuraxial anaesthesia [3].

There are several options for administering neuraxial anesthesia, however, we chose a catheter-based technique due to the potential length of surgery, but also to gently titrate the surgical level and avoid cardiovascular and respiratory compromise which could necessitate emergent intubation. More specifically, we chose to utilize the dural-puncture epidural technique (DPE) as it has been shown to improve neuraxial block quality when compared to conventional epidural techniques [4].

Our patient had a significant PPH requiring transfusion. If PPH is uncontrolled, anesthetic level is not achieved or other such complications arise, patients may need to be emergently intubated and placed under general anesthesia. Obstetric patients are known to have otherwise more difficult airways [5]. This occurs due to the anatomical and physiological changes of pregnancy including upper airway edema, enlarged breasts making laryngoscopy difficult, decreased functional residual capacity with increased oxygen consumption, and reduced esophageal sphincter tone leading to a higher risk of aspiration. Emergent scenarios can exacerbate the rate of failed intubations due to the time pressure of fetus delivery, poor planning, communication, and performance. In our patient, with extensive airway history and otherwise non-reassuring airway features, intubation could have been extremely challenging. To prevent a potentially disastrous situation, electively securing and maintaining her airway with conscious sedation allowed for easy conversion to general anesthesia if that became necessary. 

Gentle titration of a remifentanil infusion allowed for ETT tolerance throughout the surgery. We chose remifentanil as it has a very short context-sensitive half-time allowing it to be easily titrated. Furthermore, it has been shown to be safe for both mothers and neonates, however, its use can cause neonatal respiratory depression [6]. As such, a high-risk neonatal resuscitation team was present in the operating room in the event the neonate required respiratory support. Its use in this case allowed the patient to tolerate initial placement and continued intubation throughout delivery.

## Conclusions

This case describes the role of prophylactic fiberoptic intubation in obstetric patients with anticipated difficult airways while utilizing neuraxial anesthesia as the primary technique. This unique method of anesthetic management can provide appropriate anesthesia for cesarean delivery while limiting potentially fatal airway compromise if complications arise.
